# Crystal structure of 3-(2-bromo­phenyl­sulfon­yl)-2,5,7-trimethyl-1-benzo­furan

**DOI:** 10.1107/S1600536814021850

**Published:** 2014-10-08

**Authors:** Hong Dae Choi, Uk Lee

**Affiliations:** a Department of Chemistry, Dongeui University, San 24 Kaya-dong, Busanjin-gu, Busan 614-714, Republic of Korea; b Department of Chemistry, Pukyong National University, 599-1 Daeyeon 3-dong, Nam-gu, Busan 608-737, Republic of Korea

**Keywords:** crystal structure, benzo­furan, 2-bromo­phen­yl, C—H⋯π hydrogen bonds, π–π inter­actions, Br⋯O contact

## Abstract

In the title compound, C_17_H_15_BrO_3_S, the dihedral angle between the planes of the benzo­furan ring system [r.m.s. deviation = 0.016 (2) Å] and the 2-bromo­phenyl ring is 82.93 (6)°. In the crystal, mol­ecules are linked *via* pairs of C—H⋯π hydrogen bonds and π–π inter­actions between the benzene and furan rings of neighbouring mol­ecules [centroid–centroid distance = 3.881 (2) Å] into inversion-related dimers along the *b*-axis direction. These dimers are further linked by short Br⋯O [3.185 (2) Å] contacts.

## Related literature   

For a related structure and background to benzo­furan derivatives, see: Choi & Lee (2014 ▶). For further synthetic details, see: Choi *et al.* (1999 ▶). For a review of halogen bonding, see: Politzer *et al.* (2007 ▶).
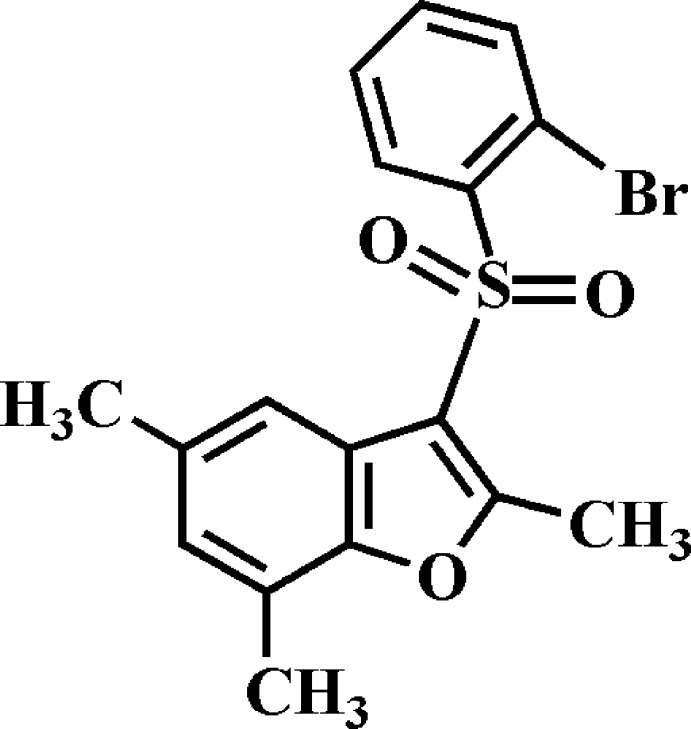


## Experimental   

### Crystal data  

C_17_H_15_BrO_3_S
*M* *_r_* = 379.26
Monoclinic, 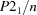

*a* = 7.8969 (2) Å
*b* = 8.1489 (2) Å
*c* = 24.3146 (6) Å
β = 96.210 (1)°
*V* = 1555.49 (7) Å^3^
*Z* = 4
Mo *K*α radiation
μ = 2.79 mm^−1^
*T* = 173 K
0.31 × 0.27 × 0.13 mm

### Data collection  

Bruker SMART APEXII CCD diffractometer
Absorption correction: multi-scan (*SADABS*; Bruker, 2009 ▶) *T*_min_ = 0.479, *T*_max_ = 0.713
27395 measured reflections
3867 independent reflections
3130 reflections with *I* > 2σ(*I*)
*R*_int_ = 0.042

### Refinement  

*R*[*F* ^2^ > 2σ(*F* ^2^)] = 0.031
*wR*(*F* ^2^) = 0.080
*S* = 1.03
3867 reflections
202 parameters
H-atom parameters constrained
Δρ_max_ = 0.51 e Å^−3^
Δρ_min_ = −0.34 e Å^−3^

### 

Data collection: *APEX2* (Bruker, 2009 ▶); cell refinement: *SAINT* (Bruker, 2009 ▶); data reduction: *SAINT*; program(s) used to solve structure: *SHELXS97* (Sheldrick, 2008 ▶); program(s) used to refine structure: *SHELXL97* (Sheldrick, 2008 ▶); molecular graphics: *ORTEP-3* for Windows (Farrugia, 2012 ▶) and *DIAMOND* (Brandenburg, 1998 ▶); software used to prepare material for publication: *SHELXL97*.

## Supplementary Material

Crystal structure: contains datablock(s) I. DOI: 10.1107/S1600536814021850/mw2127sup1.cif

Structure factors: contains datablock(s) I. DOI: 10.1107/S1600536814021850/mw2127Isup2.hkl

Supporting information file. DOI: 10.1107/S1600536814021850/mw2127Isup3.cml

**Fig. 1.**
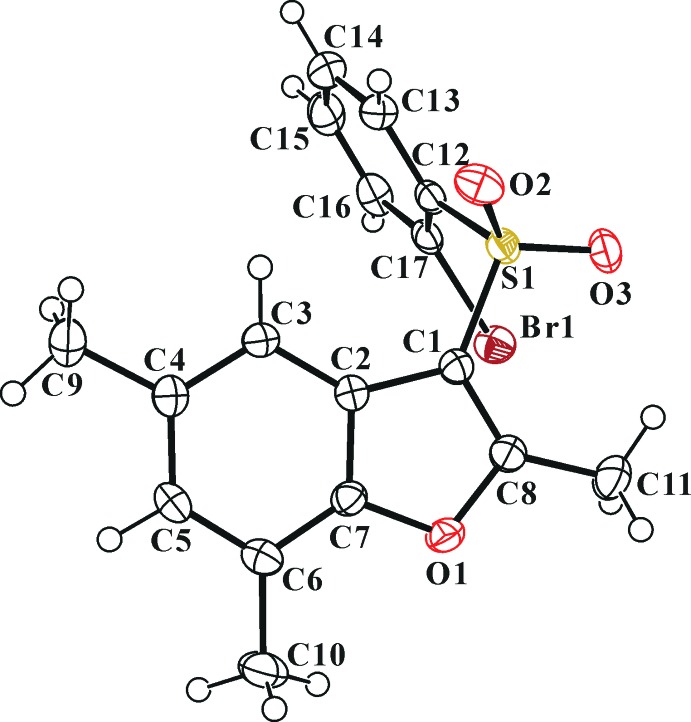
The molecular structure of the title compound with the atom numbering scheme. Displacement ellipsoids are drawn at the 50% probability level. H atoms are presented as small spheres of arbitrary radius.

. DOI: 10.1107/S1600536814021850/mw2127fig1.tif
The mol­ecular structure of the title compound with the atom numbering scheme. Displacement ellipsoids are drawn at the 50% probability level. H atoms are presented as small spheres of arbitrary radius.

**Fig. 2.**
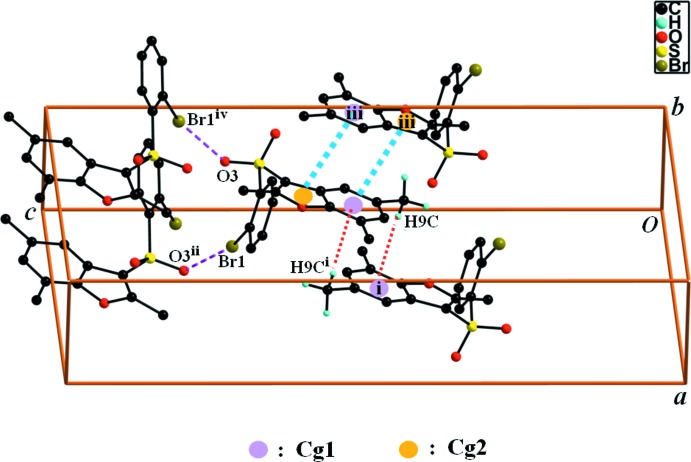
A view of the C—H···π, π–π and Br···O interactions (dotted lines) in the crystal structure of the title compound. H atoms non-participating in hydrogen-bonding were omitted for clarity. [Symmetry codes: (i) - x + 1, - y + 1, - z + 1; (ii) - x + 1/2, y - 1/2, - z + 3/2 ; (iii) - x, - y + 1, - z + 1; (iv) - x + 1/2, y + 1/2, - z + 3/2.]

Click here for additional data file.x y z x y z x y z x y z . DOI: 10.1107/S1600536814021850/mw2127fig2.tif
A view of the C—H⋯π, π–π and Br⋯O inter­actions (dotted lines) in the crystal structure of the title compound. H atoms non-participating in hydrogen-bonding were omitted for clarity. [Symmetry codes: (i) − *x* + 1, − *y* + 1, − *z* + 1; (ii) − *x* + 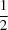, *y* − 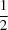, − *z* + 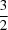; (iii) − *x*, − *y* + 1, − *z* + 1; (iv) − *x* + 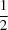, *y* + 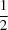, − *z* + 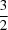.]

CCDC reference: 1027593

Additional supporting information:  crystallographic information; 3D view; checkCIF report

## Figures and Tables

**Table 1:** Hydrogen-bond geometry (, ) *Cg*1 is the centroid of the C2C7 benzene ring.

*D*H*A*	*D*H	H*A*	*D* *A*	*D*H*A*
C9H9*C* *Cg*1^i^	0.98	2.84	3.608(2)	136

Symmetry code: (i) 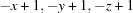.

## supplementary crystallographic information

### S1. Comment

As a part of our continuing program for benzofuran derivatives (Choi & Lee, 2014), we report herein on the crystal structure of the title compound.

In the title molecule (Fig. 1), the benzofuran unit is essentially planar, with a mean deviation of 0.016 (2) Å from the least-squares plane defined by the nine constituent atoms. The 2-bromophenyl ring is essentially planar, with a mean deviation of 0.004 (2) Å from the least-squares plane defined by the six constituent atoms. The dihedral angle formed by the benzofuran ring and the 2-bromophenyl ring is 82.93 (6)°. In the crystal structure (Fig. 2), molecules are linked *via* two different pairs of C—H···π hydrogen bonds (Table 1, Cg1 is the centroid of the C2–C7 benzene ring) and π–π interactions between the benzene and furan rings of neighbouring molecules, with a Cg1···Cg2^iii^ distance of 3.881 (2) Å and an interplanar distance of 3.562 (2) Å resulting in a slippage of 1.541 (2) Å (Cg2 is the centroid of the C1/C2/C7/O1/C8 furan ring), into inversion-related dimers along the *b*-axis direction. These dimers are further linked by halogen-bondings (Politzer *et al.* 2007) between the bromine and the O atom of the O═S═O unit [Br1..O3^ii^ = 3.185 (2) Å, C17—Br1···O3^ii^ = 164.49 (7)°, symmetry code: (ii) - *x* + 1/2, *y* - 1/2, - *z* + 3/2].

### S2. Experimental

The starting material 3-(2-bromophenylsulfanyl)-2,5,7-trimethyl-1-benzofuran was prepared by a literature method (Choi *et al.*, 1999). 3-Chloroperoxybenzoic acid (77%, 403 mg, 1.8 mmol) was added in small portions to a stirred solution of 3-(2-bromophenylsulfanyl)-2,5,7-trimethyl-1-benzofuran (278 mg, 0.8 mmol) in dichloromethane (30 ml) at 273 K. After being stirred at room temperature for 8h, the mixture was washed with saturated sodium bicarbonate solution (2 X 10 ml) and the organic layer was separated, dried over magnesium sulfate, filtered and concentrated at reduced pressure. The residue was purified by column chromatography (hexane-ethyl acetate, 4:1 *v*/*v*) to afford the title compound as a colorless solid [yield 72% (218 mg); m.p. 453–454 K; *R*_f_ = 0.51 (hexane-ethyl acetate, 4:1 *v*/*v*)]. Single crystals suitable for X-ray diffraction were prepared by slow evaporation of a solution of the title compound (21 mg) in acetone (15 ml) at room temperature.

### S3. Refinement

All H atoms were positioned geometrically and refined using a riding model, with C—H = 0.95 Å for aryl, and 0.98 Å for methyl H atoms, respectively. *U*_iso_ (H) = 1.2*U*_eq_ (C) for aryl and 1.5*U*_eq_ (C)) for methyl H atoms. The positions of methyl hydrogens were optimized using the SHELXL-97's command AFIX 137 (Sheldrick, 2008).

### Crystal data

**Table:**

C_17_H_15_BrO_3_S	*F*(000) = 768
*M**_r_* = 379.26	*D*_x_ = 1.619 Mg m^−^^3^
Monoclinic, *P*2_1_/*n*	Melting point = 454–453 K
Hall symbol: -P 2yn	Mo *K*α radiation, λ = 0.71073 Å
*a* = 7.8969 (2) Å	Cell parameters from 7387 reflections
*b* = 8.1489 (2) Å	θ = 2.6–26.1°
*c* = 24.3146 (6) Å	µ = 2.79 mm^−^^1^
β = 96.210 (1)°	*T* = 173 K
*V* = 1555.49 (7) Å^3^	Block, colourless
*Z* = 4	0.31 × 0.27 × 0.13 mm

### Data collection

**Table:**

Bruker SMART APEXII CCD diffractometer	3867 independent reflections
Radiation source: rotating anode	3130 reflections with *I* > 2σ(*I*)
Graphite multilayer monochromator	*R*_int_ = 0.042
Detector resolution: 10.0 pixels mm^-1^	θ_max_ = 28.3°, θ_min_ = 1.7°
φ and ω scans	*h* = −10→10
Absorption correction: multi-scan (*SADABS*; Bruker, 2009)	*k* = −10→10
*T*_min_ = 0.479, *T*_max_ = 0.713	*l* = −31→32
27395 measured reflections	

### Refinement

**Table:**

Refinement on *F*^2^	Primary atom site location: structure-invariant direct methods
Least-squares matrix: full	Secondary atom site location: difference Fourier map
*R*[*F*^2^ > 2σ(*F*^2^)] = 0.031	Hydrogen site location: difference Fourier map
*wR*(*F*^2^) = 0.080	H-atom parameters constrained
*S* = 1.03	*w* = 1/[σ^2^(*F*_o_^2^) + (0.0401*P*)^2^ + 0.8758*P*] where *P* = (*F*_o_^2^ + 2*F*_c_^2^)/3
3867 reflections	(Δ/σ)_max_ = 0.001
202 parameters	Δρ_max_ = 0.51 e Å^−^^3^
0 restraints	Δρ_min_ = −0.34 e Å^−^^3^

### Special details

**Table:**

Experimental. ^1^H NMR (δ p.p.m., CDCl_3_, 400 Hz): 8.49 (d, J = 6.12 Hz, 1H), 7.65 (d, J =6.84 Hz, 1H), 7.53-7.58 (m, 1H), 7.38-7.43 (m, 1H), 7.06 (s, 1H), 6.88 (s, 1H), 2.84 (s, 3H), 2.43 (s, 3H), 2.31 (s, 3H).
Geometry. All esds (except the esd in the dihedral angle between two l.s. planes) are estimated using the full covariance matrix. The cell esds are taken into account individually in the estimation of esds in distances, angles and torsion angles; correlations between esds in cell parameters are only used when they are defined by crystal symmetry. An approximate (isotropic) treatment of cell esds is used for estimating esds involving l.s. planes.
Refinement. Refinement of F^2^ against ALL reflections. The weighted R-factor wR and goodness of fit S are based on F^2^, conventional R-factors R are based on F, with F set to zero for negative F^2^. The threshold expression of F^2^ > 2sigma(F^2^) is used only for calculating R-factors(gt) etc. and is not relevant to the choice of reflections for refinement. R-factors based on F^2^ are statistically about twice as large as those based on F, and R- factors based on ALL data will be even larger.

### Fractional atomic coordinates and isotropic or equivalent isotropic displacement parameters (Å^2^)

**Table:**

	*x*	*y*	*z*	*U*_iso_*/*U*_eq_	
Br1	0.46256 (3)	0.42412 (3)	0.713662 (9)	0.03093 (9)	
S1	0.12638 (7)	0.65915 (7)	0.65837 (2)	0.02299 (13)	
O1	0.1202 (2)	0.22941 (18)	0.58642 (6)	0.0280 (4)	
O2	0.0262 (2)	0.7852 (2)	0.62933 (7)	0.0320 (4)	
O3	0.0830 (2)	0.6094 (2)	0.71169 (7)	0.0311 (4)	
C1	0.1294 (3)	0.4896 (3)	0.61574 (9)	0.0222 (4)	
C2	0.1822 (3)	0.4890 (3)	0.56071 (8)	0.0208 (4)	
C3	0.2360 (3)	0.6058 (3)	0.52431 (9)	0.0241 (4)	
H3	0.2402	0.7190	0.5337	0.029*	
C4	0.2832 (3)	0.5521 (3)	0.47408 (9)	0.0257 (5)	
C5	0.2767 (3)	0.3855 (3)	0.46099 (9)	0.0273 (5)	
H5	0.3106	0.3521	0.4264	0.033*	
C6	0.2235 (3)	0.2660 (3)	0.49579 (9)	0.0270 (5)	
C7	0.1761 (3)	0.3251 (3)	0.54484 (9)	0.0229 (4)	
C8	0.0946 (3)	0.3321 (3)	0.62932 (9)	0.0258 (5)	
C9	0.3440 (3)	0.6725 (3)	0.43345 (10)	0.0350 (6)	
H9A	0.2662	0.7664	0.4294	0.053*	
H9B	0.3467	0.6188	0.3975	0.053*	
H9C	0.4587	0.7105	0.4470	0.053*	
C10	0.2218 (4)	0.0869 (3)	0.48191 (12)	0.0412 (6)	
H10A	0.2464	0.0228	0.5159	0.062*	
H10B	0.3084	0.0642	0.4570	0.062*	
H10C	0.1093	0.0566	0.4638	0.062*	
C11	0.0392 (3)	0.2511 (3)	0.67846 (10)	0.0378 (6)	
H11A	0.1317	0.1821	0.6958	0.057*	
H11B	−0.0608	0.1827	0.6674	0.057*	
H11C	0.0099	0.3344	0.7049	0.057*	
C12	0.3401 (3)	0.7318 (3)	0.66497 (8)	0.0220 (4)	
C13	0.3630 (3)	0.8918 (3)	0.64809 (10)	0.0286 (5)	
H13	0.2678	0.9543	0.6327	0.034*	
C14	0.5241 (3)	0.9611 (3)	0.65352 (11)	0.0365 (6)	
H14	0.5390	1.0714	0.6423	0.044*	
C15	0.6626 (3)	0.8704 (3)	0.67508 (11)	0.0363 (6)	
H15	0.7730	0.9181	0.6785	0.044*	
C16	0.6417 (3)	0.7101 (3)	0.69174 (10)	0.0315 (5)	
H16	0.7377	0.6476	0.7063	0.038*	
C17	0.4809 (3)	0.6409 (3)	0.68725 (9)	0.0238 (4)	

### Atomic displacement parameters (Å^2^)

**Table:**

	*U*^11^	*U*^22^	*U*^33^	*U*^12^	*U*^13^	*U*^23^
Br1	0.03549 (14)	0.02720 (13)	0.02903 (14)	0.00525 (10)	−0.00148 (9)	0.00300 (9)
S1	0.0223 (3)	0.0245 (3)	0.0227 (3)	0.0008 (2)	0.0050 (2)	−0.0029 (2)
O1	0.0390 (9)	0.0201 (7)	0.0247 (8)	−0.0047 (7)	0.0030 (7)	0.0020 (6)
O2	0.0277 (8)	0.0306 (9)	0.0366 (9)	0.0082 (7)	−0.0009 (7)	−0.0044 (7)
O3	0.0319 (8)	0.0388 (10)	0.0243 (8)	−0.0031 (7)	0.0113 (7)	−0.0042 (7)
C1	0.0231 (10)	0.0228 (10)	0.0209 (11)	−0.0007 (8)	0.0030 (8)	−0.0005 (8)
C2	0.0197 (10)	0.0225 (10)	0.0201 (10)	0.0011 (8)	0.0018 (8)	0.0005 (8)
C3	0.0275 (11)	0.0204 (10)	0.0244 (11)	−0.0008 (8)	0.0033 (9)	−0.0006 (8)
C4	0.0248 (11)	0.0295 (11)	0.0229 (11)	0.0006 (9)	0.0031 (9)	0.0018 (9)
C5	0.0304 (11)	0.0307 (12)	0.0214 (11)	0.0033 (9)	0.0052 (9)	−0.0048 (9)
C6	0.0299 (11)	0.0255 (11)	0.0248 (12)	0.0019 (9)	−0.0001 (9)	−0.0043 (9)
C7	0.0258 (11)	0.0208 (10)	0.0217 (11)	−0.0016 (8)	0.0012 (8)	0.0016 (8)
C8	0.0282 (11)	0.0258 (11)	0.0236 (11)	−0.0030 (9)	0.0031 (9)	−0.0012 (9)
C9	0.0391 (14)	0.0392 (14)	0.0283 (13)	−0.0014 (11)	0.0106 (11)	0.0039 (11)
C10	0.0618 (18)	0.0252 (12)	0.0368 (15)	0.0020 (12)	0.0065 (13)	−0.0082 (11)
C11	0.0508 (16)	0.0351 (13)	0.0288 (13)	−0.0102 (12)	0.0098 (11)	0.0050 (11)
C12	0.0242 (10)	0.0244 (11)	0.0180 (10)	−0.0011 (8)	0.0055 (8)	−0.0046 (8)
C13	0.0346 (12)	0.0241 (11)	0.0278 (12)	0.0005 (9)	0.0057 (10)	−0.0006 (9)
C14	0.0477 (15)	0.0286 (12)	0.0349 (14)	−0.0092 (11)	0.0124 (12)	−0.0037 (10)
C15	0.0308 (12)	0.0455 (15)	0.0339 (14)	−0.0116 (11)	0.0085 (11)	−0.0094 (12)
C16	0.0256 (11)	0.0428 (14)	0.0261 (12)	0.0003 (10)	0.0034 (9)	−0.0053 (10)
C17	0.0293 (11)	0.0262 (11)	0.0163 (10)	0.0023 (9)	0.0044 (8)	−0.0027 (8)

### Geometric parameters (Å, º)

**Table:**

Br1—C17	1.890 (2)	C8—C11	1.472 (3)
Br1—O3^i^	22.6093 (17)	C9—H9A	0.9800
S1—O3	1.4345 (16)	C9—H9B	0.9800
S1—O2	1.4348 (17)	C9—H9C	0.9800
S1—C1	1.729 (2)	C10—H10A	0.9800
S1—C12	1.779 (2)	C10—H10B	0.9800
O1—C8	1.369 (3)	C10—H10C	0.9800
O1—C7	1.386 (3)	C11—H11A	0.9800
C1—C8	1.361 (3)	C11—H11B	0.9800
C1—C2	1.444 (3)	C11—H11C	0.9800
C2—C7	1.390 (3)	C12—C13	1.385 (3)
C2—C3	1.397 (3)	C12—C17	1.396 (3)
C3—C4	1.386 (3)	C13—C14	1.385 (4)
C3—H3	0.9500	C13—H13	0.9500
C4—C5	1.395 (3)	C14—C15	1.376 (4)
C4—C9	1.507 (3)	C14—H14	0.9500
C5—C6	1.385 (3)	C15—C16	1.383 (4)
C5—H5	0.9500	C15—H15	0.9500
C6—C7	1.375 (3)	C16—C17	1.383 (3)
C6—C10	1.497 (3)	C16—H16	0.9500
			
C17—Br1—O3^i^	76.20 (6)	H9A—C9—H9B	109.5
O3—S1—O2	118.32 (10)	C4—C9—H9C	109.5
O3—S1—C1	109.64 (10)	H9A—C9—H9C	109.5
O2—S1—C1	108.65 (10)	H9B—C9—H9C	109.5
O3—S1—C12	109.22 (10)	C6—C10—H10A	109.5
O2—S1—C12	105.85 (10)	C6—C10—H10B	109.5
C1—S1—C12	104.19 (10)	H10A—C10—H10B	109.5
C8—O1—C7	107.27 (16)	C6—C10—H10C	109.5
C8—C1—C2	107.93 (19)	H10A—C10—H10C	109.5
C8—C1—S1	126.30 (17)	H10B—C10—H10C	109.5
C2—C1—S1	125.62 (16)	C8—C11—H11A	109.5
C7—C2—C3	118.87 (19)	C8—C11—H11B	109.5
C7—C2—C1	104.80 (18)	H11A—C11—H11B	109.5
C3—C2—C1	136.3 (2)	C8—C11—H11C	109.5
C4—C3—C2	118.2 (2)	H11A—C11—H11C	109.5
C4—C3—H3	120.9	H11B—C11—H11C	109.5
C2—C3—H3	120.9	C13—C12—C17	119.4 (2)
C3—C4—C5	120.1 (2)	C13—C12—S1	116.07 (17)
C3—C4—C9	120.5 (2)	C17—C12—S1	124.50 (17)
C5—C4—C9	119.4 (2)	C12—C13—C14	120.3 (2)
C6—C5—C4	123.5 (2)	C12—C13—H13	119.9
C6—C5—H5	118.3	C14—C13—H13	119.9
C4—C5—H5	118.3	C15—C14—C13	120.1 (2)
C7—C6—C5	114.4 (2)	C15—C14—H14	120.0
C7—C6—C10	122.7 (2)	C13—C14—H14	120.0
C5—C6—C10	122.9 (2)	C14—C15—C16	120.3 (2)
C6—C7—O1	125.00 (19)	C14—C15—H15	119.9
C6—C7—C2	124.9 (2)	C16—C15—H15	119.9
O1—C7—C2	110.03 (18)	C17—C16—C15	120.0 (2)
C1—C8—O1	109.95 (18)	C17—C16—H16	120.0
C1—C8—C11	134.9 (2)	C15—C16—H16	120.0
O1—C8—C11	115.2 (2)	C16—C17—C12	120.0 (2)
C4—C9—H9A	109.5	C16—C17—Br1	117.25 (18)
C4—C9—H9B	109.5	C12—C17—Br1	122.73 (17)
			
O3—S1—C1—C8	0.8 (2)	C1—C2—C7—O1	1.1 (2)
O2—S1—C1—C8	−129.9 (2)	C2—C1—C8—O1	−0.4 (2)
C12—S1—C1—C8	117.6 (2)	S1—C1—C8—O1	−176.16 (16)
O3—S1—C1—C2	−174.23 (18)	C2—C1—C8—C11	178.8 (3)
O2—S1—C1—C2	55.1 (2)	S1—C1—C8—C11	3.0 (4)
C12—S1—C1—C2	−57.4 (2)	C7—O1—C8—C1	1.1 (2)
C8—C1—C2—C7	−0.4 (2)	C7—O1—C8—C11	−178.3 (2)
S1—C1—C2—C7	175.34 (16)	O3—S1—C12—C13	−121.32 (17)
C8—C1—C2—C3	−178.9 (2)	O2—S1—C12—C13	7.10 (19)
S1—C1—C2—C3	−3.1 (4)	C1—S1—C12—C13	121.60 (18)
C7—C2—C3—C4	−0.8 (3)	O3—S1—C12—C17	56.5 (2)
C1—C2—C3—C4	177.4 (2)	O2—S1—C12—C17	−175.07 (17)
C2—C3—C4—C5	−0.1 (3)	C1—S1—C12—C17	−60.6 (2)
C2—C3—C4—C9	−179.4 (2)	C17—C12—C13—C14	−0.3 (3)
C3—C4—C5—C6	0.4 (4)	S1—C12—C13—C14	177.65 (18)
C9—C4—C5—C6	179.7 (2)	C12—C13—C14—C15	0.8 (4)
C4—C5—C6—C7	0.3 (3)	C13—C14—C15—C16	−0.4 (4)
C4—C5—C6—C10	−178.2 (2)	C14—C15—C16—C17	−0.6 (4)
C5—C6—C7—O1	−179.3 (2)	C15—C16—C17—C12	1.2 (3)
C10—C6—C7—O1	−0.8 (4)	C15—C16—C17—Br1	−177.56 (18)
C5—C6—C7—C2	−1.4 (3)	C13—C12—C17—C16	−0.7 (3)
C10—C6—C7—C2	177.2 (2)	S1—C12—C17—C16	−178.47 (17)
C8—O1—C7—C6	176.8 (2)	C13—C12—C17—Br1	177.95 (16)
C8—O1—C7—C2	−1.4 (2)	S1—C12—C17—Br1	0.2 (3)
C3—C2—C7—C6	1.7 (3)	O3^i^—Br1—C17—C16	−99.99 (17)
C1—C2—C7—C6	−177.1 (2)	O3^i^—Br1—C17—C12	81.32 (17)
C3—C2—C7—O1	179.89 (18)		

Symmetry code: (i) −*x*+1/2, *y*−1/2, −*z*+1/2.

### Hydrogen-bond geometry (Å, º)

Cg1 is the centroid of the C2–C7 benzene ring.

**Table:**

*D*—H···*A*	*D*—H	H···*A*	*D*···*A*	*D*—H···*A*
C9—H9*C*···*Cg*1^ii^	0.98	2.84	3.608 (2)	136

Symmetry code: (ii) −*x*+1, −*y*+1, −*z*+1.
