# Supplementary figures and images for: Crystal structure of 3-(2-bromo­phenyl­sulfon­yl)-2,5,7-trimethyl-1-benzo­furan
Source: Acta Crystallogr Sect E Struct Rep Online. 2014 Oct 8;70(Pt 11):o1152. doi: 10.1107/S1600536814021850 (PMC4257325; doi:10.1107/S1600536814021850)

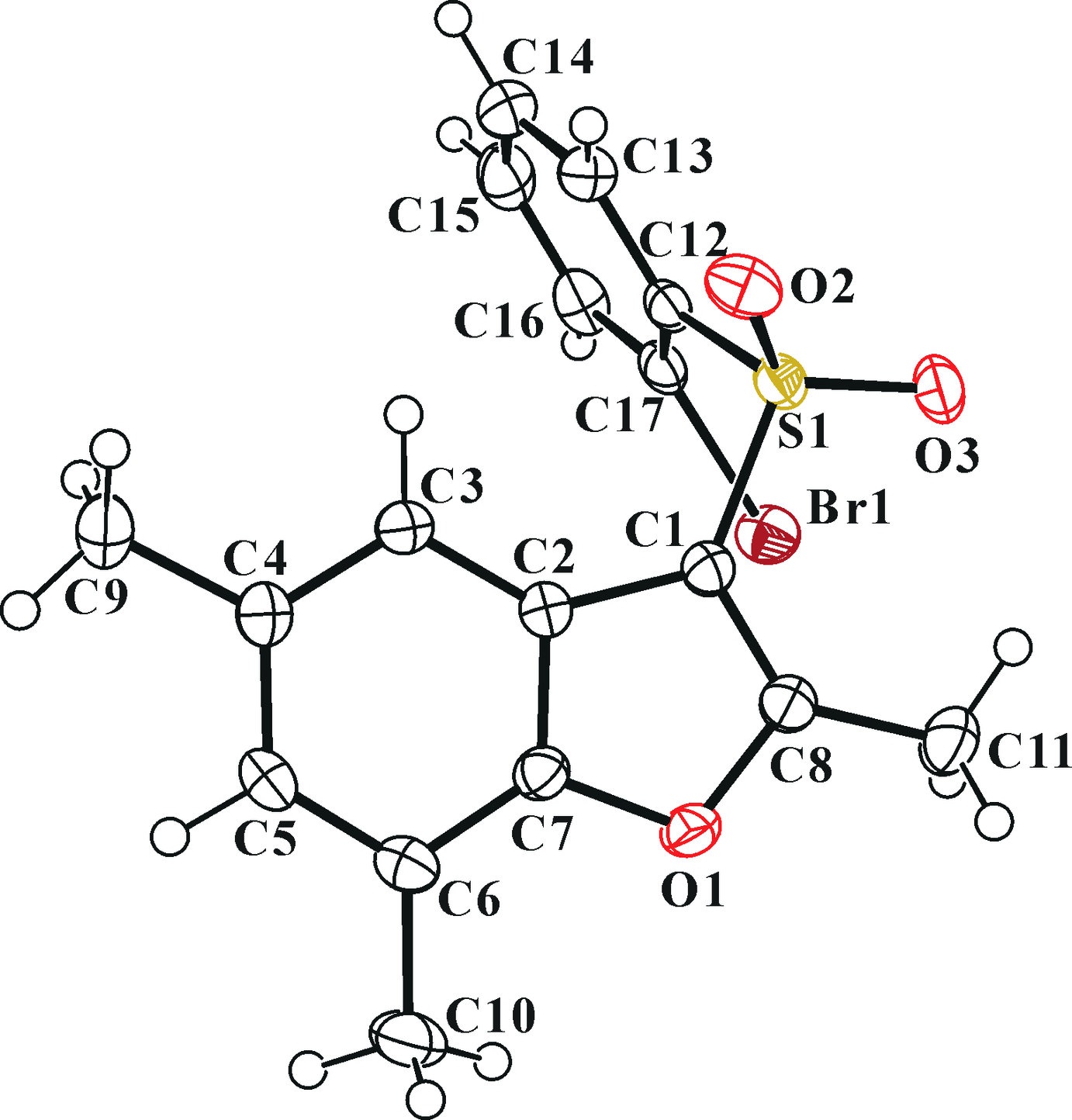

Supplement: Supplementary file 4 [file e-70-o1152-fig1.tif]

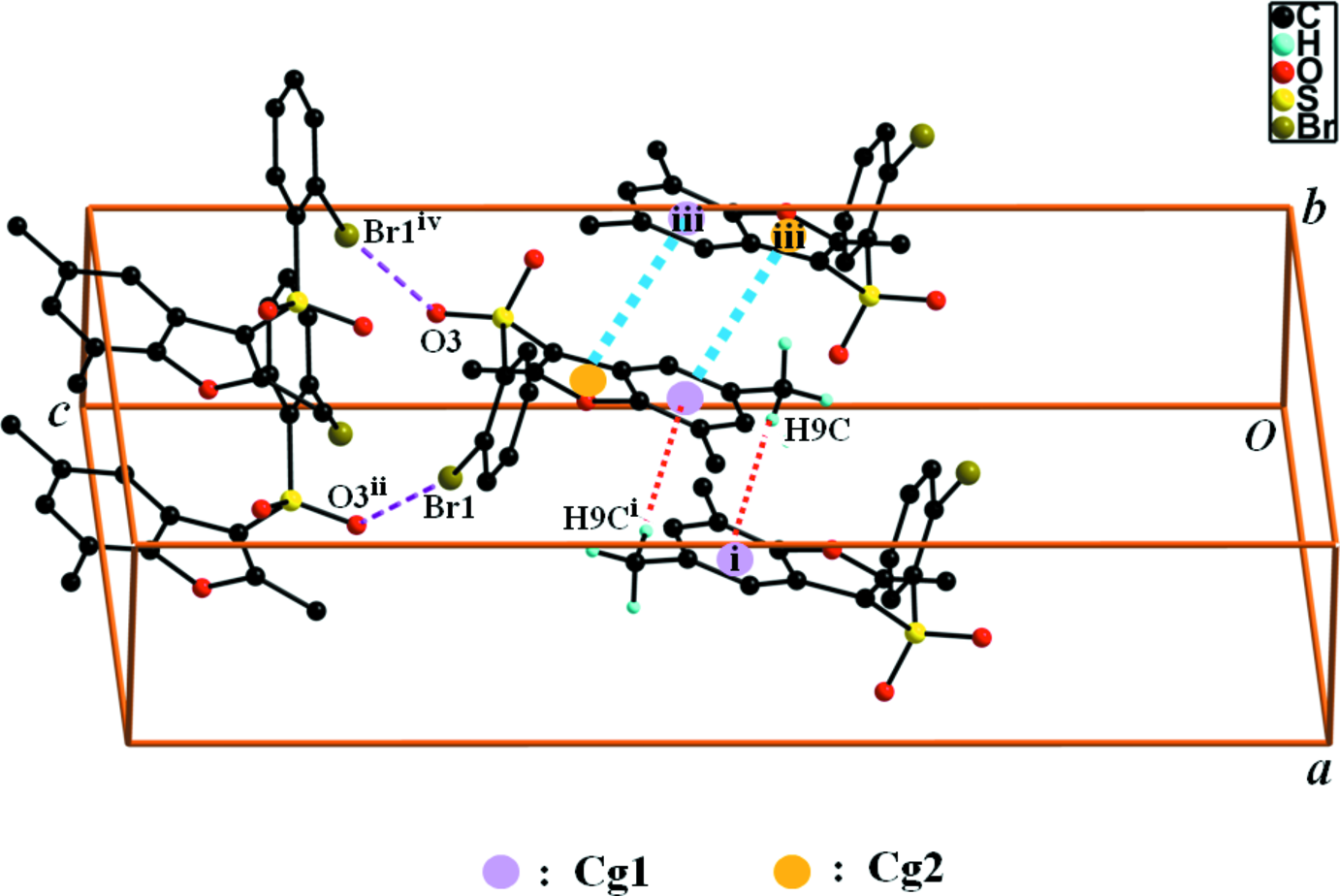

Supplement: Supplementary file 5 [file e-70-o1152-fig2.tif]
